# RNA-Seq analysis validates the use of culture-derived *Trypanosoma brucei* and provides new markers for mammalian and insect life-cycle stages

**DOI:** 10.1186/s12864-018-4600-6

**Published:** 2018-04-02

**Authors:** Arunasalam Naguleswaran, Nicholas Doiron, Isabel Roditi

**Affiliations:** Institute of Cell Biology, University of Bern, Baltzerstrasse 4, CH-3012 Bern, Switzerland

**Keywords:** Trypanosome, Transcriptome, RNA-Seq, Culture-derived, Stage-specific, Cation transporters, Amino acid transporters, Tsetse, Replacement of animal models, 3R

## Abstract

**Background:**

*Trypanosoma brucei brucei*, the parasite causing Nagana in domestic animals, is closely related to the parasites causing sleeping sickness, but does not infect humans. In addition to its importance as a pathogen, the relative ease of genetic manipulation and an innate capacity for RNAi extend its use as a model organism in cell and infection biology. During its development in its mammalian and insect (tsetse fly) hosts, *T. b. brucei* passes through several different life-cycle stages. There are currently four life-cycle stages that can be cultured: slender forms and stumpy forms, which are equivalent to forms found in the mammal, and early and late procyclic forms, which are equivalent to forms in the tsetse midgut. Early procyclic forms show coordinated group movement (social motility) on semi-solid surfaces, whereas late procyclic forms do not.

**Results:**

RNA-Seq was performed on biological replicates of each life-cycle stage. These constitute the first datasets for culture-derived slender and stumpy bloodstream forms and early and late procyclic forms. Expression profiles confirmed that genes known to be stage-regulated in the animal and insect hosts were also regulated in culture. Sequence reads of 100–125 bases provided sufficient precision to uncover differential expression of closely related genes. More than 100 transcripts showed peak expression in stumpy forms, including adenylate cyclases and several components of inositol metabolism. Early and late procyclic forms showed differential expression of 73 transcripts, a number of which encoded proteins that were previously shown to be stage-regulated. Moreover, two adenylate cyclases previously shown to reduce social motility are up-regulated in late procyclic forms.

**Conclusions:**

This study validates the use of cultured bloodstream forms as alternatives to animal-derived parasites and yields new markers for all four stages. In addition to underpinning recent findings that early and late procyclic forms are distinct life-cycle stages, it could provide insights into the reasons for their different biological properties.

**Electronic supplementary material:**

The online version of this article (10.1186/s12864-018-4600-6) contains supplementary material, which is available to authorized users.

## Background

Virtually all unicellular parasites, particularly those that depend on two hosts, progress through a series of different life-cycle stages that can differ radically in their morphology, metabolic capabilities and surface architecture. One case in point is the African trypanosome, *T. brucei brucei*, which causes the disease Nagana in cattle, and is closely related to the parasites causing human sleeping sickness.

*T. b. brucei* cycles between vertebrates and tsetse flies, the latter being the definitive hosts where sexual reproduction can take place. There are at least two morphologically distinct life-cycle stages in the mammal: the slender form, which has the capacity to replicate, and the stumpy form, which is non-dividing. Until recently it was assumed that these forms were restricted to the bloodstream, but they are now known to occur in adipose tissue and skin as well [1–3]. When parasites are taken up by a tsetse fly in the course of a blood meal, slender forms are eliminated, but stumpy forms differentiate to procyclic forms that colonise the midgut. There are two populations of procyclic forms: early procyclic forms are found for up to a week after transmission, and are positive for the surface protein GPEET procyclin, while late procyclic forms, which are responsible for persistent infection of the midgut, are GPEET-negative. These two forms cannot be distinguished by their morphology. To complete the life cycle trypanosomes must undergo several more rounds of differentiation, culminating in the delivery of infectious metacyclic forms to a new mammalian host when the tsetse takes a blood meal.

Pleomorphic stocks of *T. b. brucei*, which produce both slender and stumpy bloodstream forms, can be cultured in the presence of an extracellular matrix such as methylcellulose. In vitro, differentiation to procyclic forms is induced by the addition of citrate and/or cis-aconitate to the medium and a reduction in temperature from 37 °C to 27 °C. Procyclic culture forms initially express GPEET; depending on the culture medium they continue to grow as early procyclic forms or differentiate into late procyclic forms [4]. Glycerol, glucose, oxygen concentration and an uncharacterised midgut factor can influence GPEET expression via its 3’ UTR [4, 5]. Other factors such as serum concentration and cell density may also influence expression, but these have not been investigated systematically. In addition to GPEET, a recent study identified several transcripts and proteins that were differentially expressed in early and late procyclic forms [6]. The two life-cycle stages also showed differences in behaviour when plated on semi-solid media. Early procyclic forms exhibited social motility (SoMo), a form of coordinated group movement, while late procyclic forms replicated at the inoculation site but did not migrate [6]. In tsetse flies the progression from early to late procyclic forms is strictly unidirectional. In culture, however, differentiation/dedifferentiation can occur in both directions [5] and it is not predictable when and why this occurs.

Several previous studies have analysed the transcriptomes of different life-cycle stages of *T. b. brucei* [7, 8]. It is difficult to make comparisons, however, since some employed microarrays, while others used splice leader trapping or classical RNA-Seq. For the most part, it is also not clear whether the procyclic forms used in these studies were early, late or a mixture of the two. To obtain a more comprehensive overview of differentially expressed genes we performed RNA-Seq on defined cultures of slender bloodstream forms, stumpy bloodstream forms and early and late procyclic forms. In addition, we analysed the expression of several multigene families and found that individual members were expressed in a stage-specific manner.

## Methods

### Parasite cultivation

Pleomorphic bloodstream forms of *Trypanosoma brucei brucei* EATRO 1125, clone AnTat 1.1 [9] were originally obtained from Dr. Erik Vassella, University of Bern. Bloodstream forms cultivated in HMI-9 supplemented with 1.1% methylcellulose [10]. At densities < 10^6^ ml^− 1^, the majority of cells are slender forms; 24 h after reaching a density of 5 × 10^6^ ml^− 1^, the cells are essentially pure stumpy forms [11]. Early procyclic forms were obtained as described by Knüsel and Roditi [12]. They were maintained in SDM79 supplemented with 10% heat-inactivated foetal bovine serum in the presence of 20 mM glycerol. To differentiate them into late procyclic forms glycerol was removed from the culture medium [4]. Real-time PCR showed that the early procyclic forms expressed 26-fold more GPEET mRNA than the corresponding late procyclic forms used for RNA-Seq.

### RNA isolation and RNA-Seq analysis

Total RNA was isolated as described previously [12] and subjected to DNase treatment to remove residual genomic DNA contamination. Illumina cDNA libraries were prepared using TruSeq RNA sample preparation from a poly(A)-selected RNA. Sequencing of cDNA libraries was performed at Fasteris, Geneva, using Illumina Hiseq sequencing systems with 100 or 125 bp read lengths and sequence depths of > 40 million reads per sample. Reads were mapped to the *T. b. brucei* 927 reference genome version 5 (either coding sequences or putative 3’UTRs), using the bowtie tool available in Galaxy Interface (usegalaxy.org) with default parameters that allow a maximum of 2 mismatches per 28 bp seed (Galaxy version 1.1.2). Sequencing depth and mapping coverage are provided in Additional file 1. Mapping to the genome was used to visualise the data on Gbrowse; to estimate transcript abundance, reads were first mapped to coding sequences and unmapped reads were re-mapped to 3’ UTRs. Read counts for the annotated genes or 3’UTR were extracted using SAMTools pileup and RPM values were calculated. Bioconductor package DESeq [13] was used to identify the differentially expressed genes from biological replicates.

## Results

### Faithful expression of stage-specific markers in culture-derived trypanosomes

**Fig. 1:**
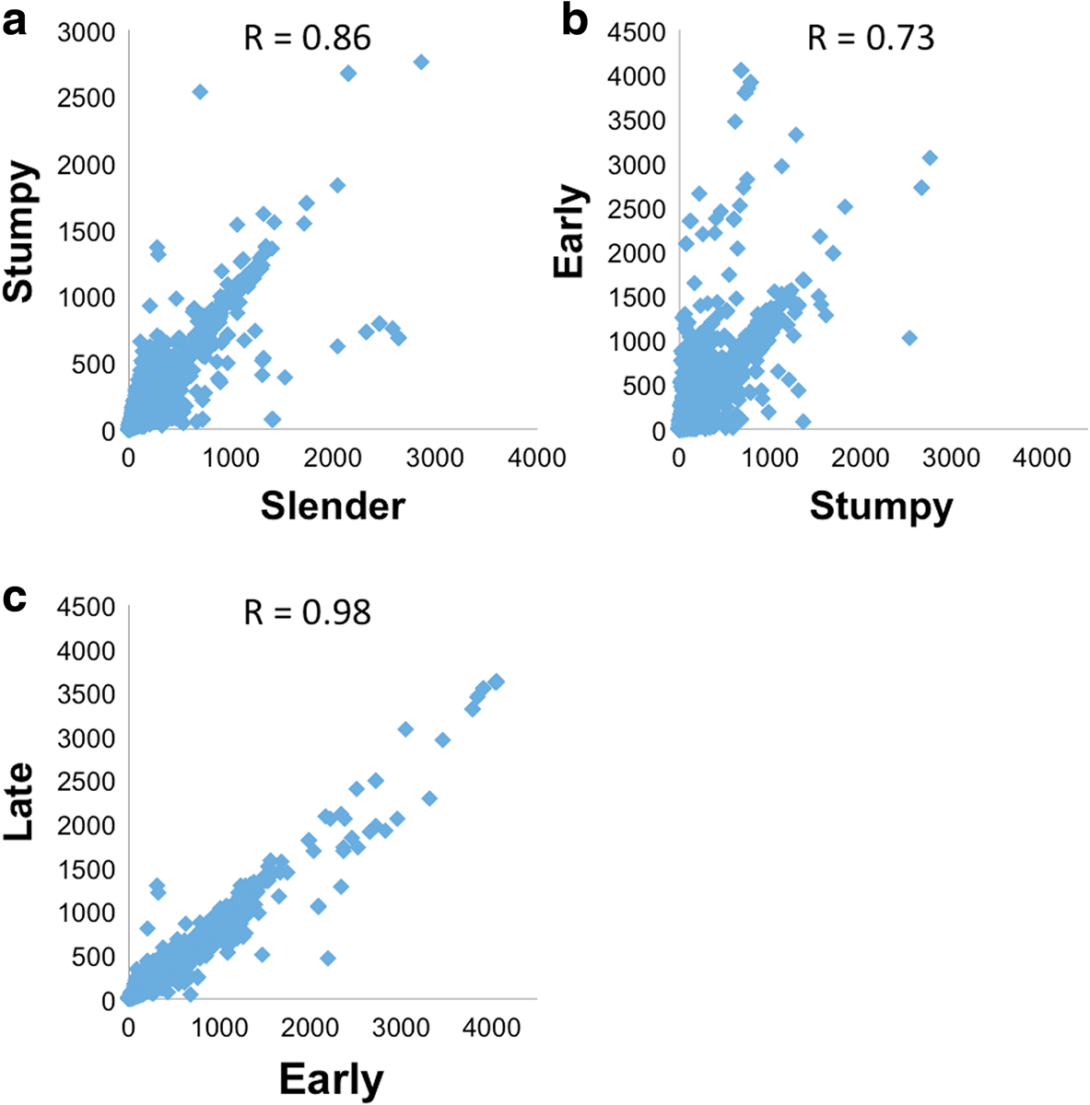
Scatter plots comparing expression profiles (reads per million) of transcripts in successive life-cycle stages. Pearson correlation values (R) are shown for each plot. Comparative transcriptomes of **a** slender vs stumpy forms, **b** stumpy vs early procyclic forms, **c** early vs late procyclic forms

**Fig. 2:**
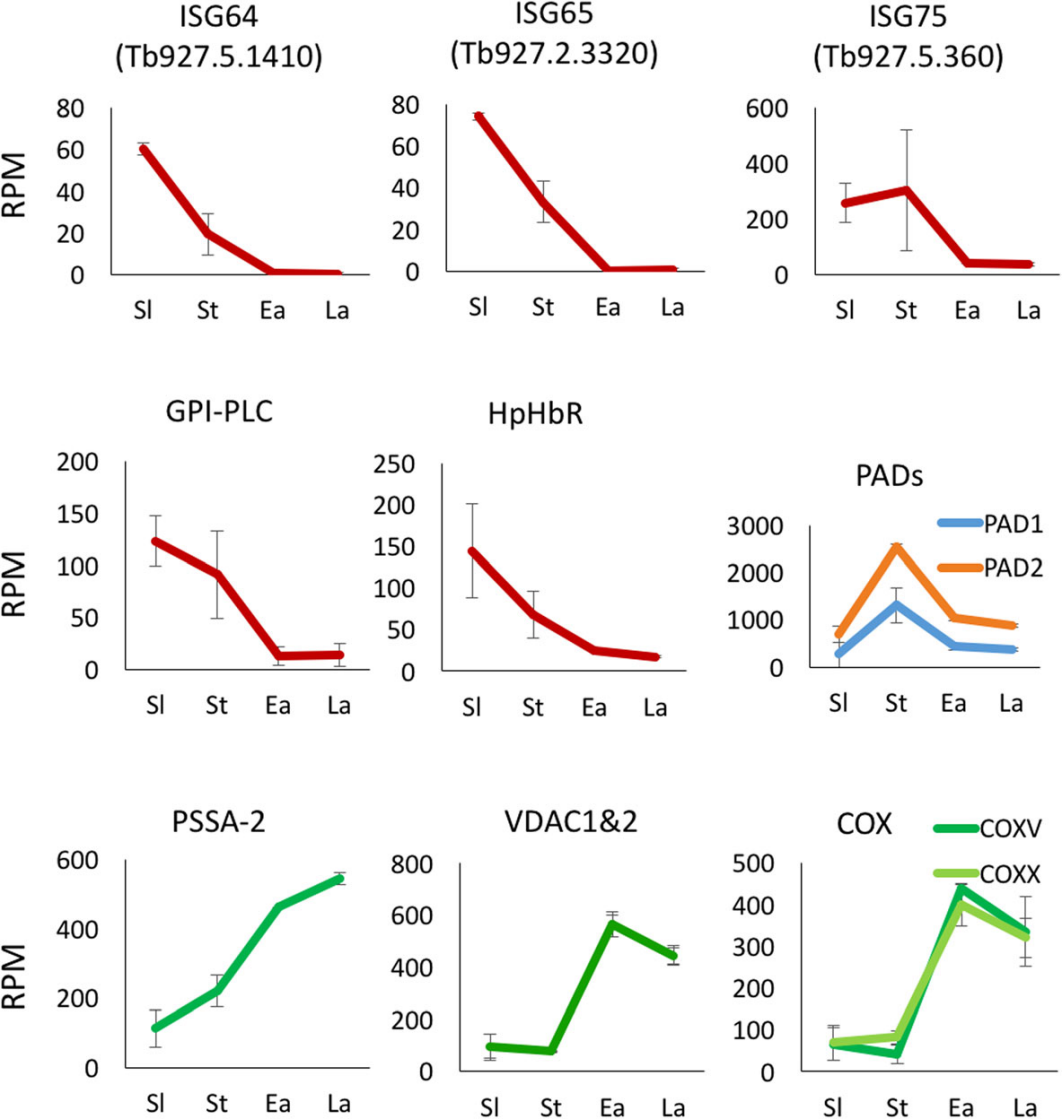
Expression profiles of known stage-regulated genes. Reads from biological replicates were mapped to annotated coding sequences. Error bars indicate standard deviations. Sl: slender bloodstream forms; St: stumpy bloodstream forms; Ea: early procyclic forms; La: late procyclic forms. RPM: reads per million

**Fig. 3:**
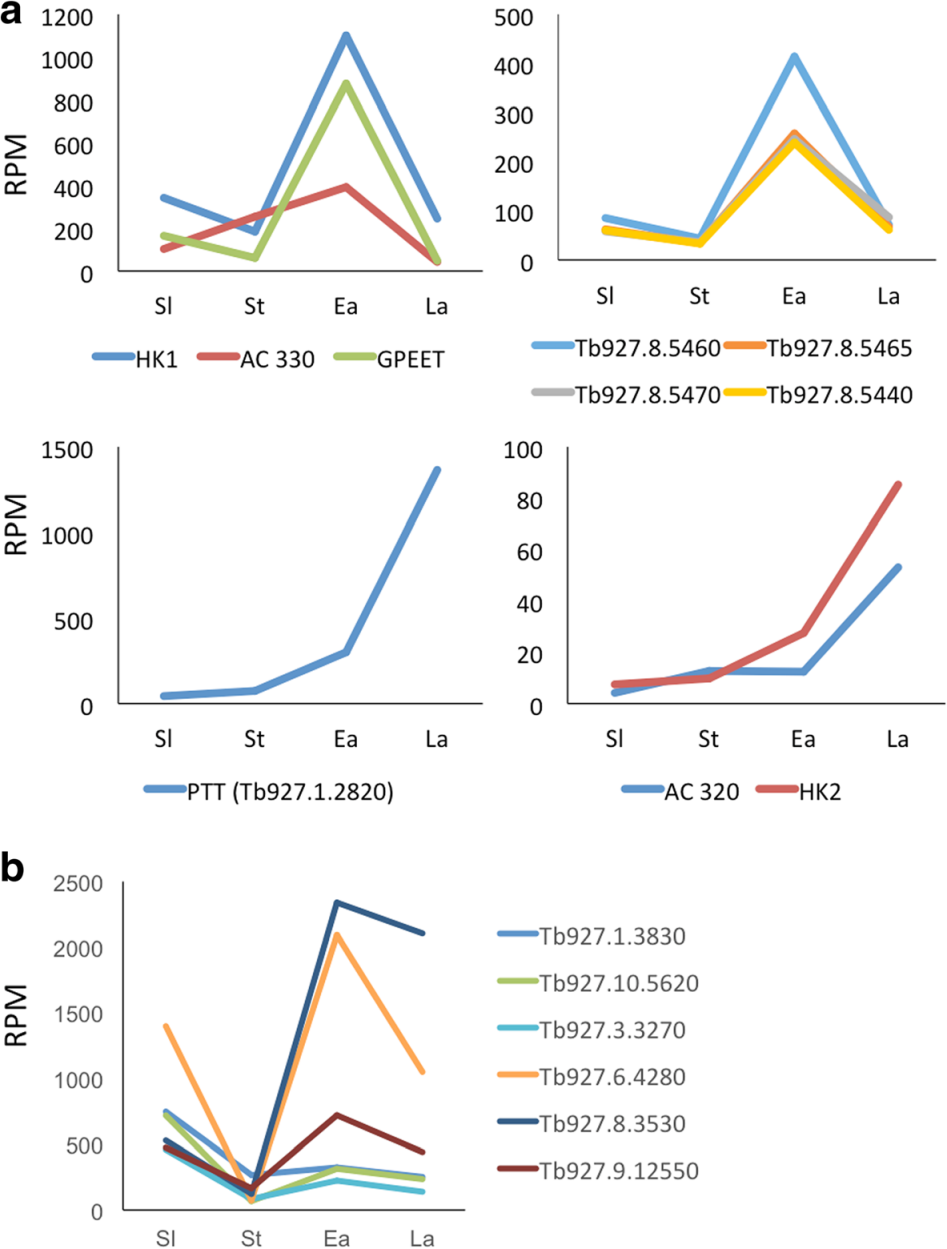
**a** Expression profiles of genes known to be differentially expressed in early and late procyclic forms. GPEET (Tb927.6.510) is the major surface protein of early procyclic forms [4]. HK1: hexokinase 1; HK2: hexokinase 2; AC320, adenylate cyclase (Tb927.5.320); AC330, adenylate cyclase (Tb927.5.330); calflagins (Tb927.8.5440, 5460, 5465 and 5470); PTT, putative pteridine transporter. Reads for HK1 and HK2 were restricted to the 3’ UTRs. **b** Down-regulation of glycosomal enzymes in stumpy forms. Tb927.1.3830, glucose-6-phosphate isomerase; Tb927.10.5620, aldolase; Tb927.3.3270, phosphofructokinase; Tb927.6.4280, GAPDH; Tb927.8.3530, glycerol-3-phosphate dehydrogenase; Tb927.9.12550, glycerol kinase 1. Sl: slender bloodstream forms; St: stumpy bloodstream forms; Ea: early procyclic forms; La: late procyclic forms. RPM: reads per million

To place our analysis of the transcriptomes of early and late procyclic forms in a wider context, we compared them to culture-derived slender and stumpy bloodstream forms of a tsetse-transmissible pleomorphic strain of *T. b. brucei.* RNA-Seq was performed on biological replicates. Pearson’s correlations for replicates of slender, stumpy, early and late procyclic forms were 0.86, 0.85, 0.95 and 0.97, respectively. When consecutive life-cycle stages were compared, the greatest differences were observed between stumpy and early procyclic forms, presumably reflecting the adaptation to a different host, followed by slender/stumpy forms (Fig. 1). We first validated our data by examining the expression profiles of a panel of genes that are known to be stage-regulated. As shown in Fig. 2 and Additional file 2, bloodstream-specific genes such as invariant surface glycoproteins ISG75, ISG65 and ISG64 [14, 15], GPI-phospholipase C (GPI-PLC) [16]; and haptoglobin-haemoglobin receptor (HpHbR) [17] are up-regulated in bloodstream forms compared to procyclic forms. Furthermore, two transcripts previously shown to accumulate in rodent-derived stumpy forms (PAD1 and PAD2) [18] showed increased expression in culture-derived stumpy forms (Fig. 2). Differentiation to the procyclic form is accompanied by expression of procyclic-specific surface proteins and development of a fully functional mitochondrion. In addition to the procyclins, which are the most abundant procyclic-specific transcripts and proteins [19], our data showed strong up-regulation of transcripts encoding the surface protein PSSA-2 [20], two voltage-dependent anion-selective channels (VDAC1 and 2) [21, 22]) and cytochrome oxidases [23] (Fig. 2). Finally, transcripts that are differentially expressed by early and late procyclic forms in both culture and tsetse [6] gave the same expression profile in our RNA-Seq data (Fig. 3a).

### The transcriptomes of culture-derived slender and stumpy forms

Pleomorphic bloodstream forms grown in HMI-9 in the presence of 1.1% methycellulose replicate as slender forms, and differentiate into stumpy forms once they reach densities > 5 × 10^6^ ml^− 1^ [11]. Stumpy forms from these cultures can differentiate synchronously to procyclic forms [10], have increased levels of PAD1 mRNA, are infectious for tsetse and can complete the life cycle [11]. As shown in Additional file 2, DESeq analysis of biological replicates identified 497 genes as differentially regulated ≥2-fold between slender forms and stumpy forms with a *p* value < 0.05. GO term analyses indicate significant decreases (Bonferroni adjusted *p* value < 10^− 5^) in transcripts in the categories of macromolecule synthesis, metabolism, chromatin assembly and locomotion (Additional file 3). Among them are 230 transcripts that were down-regulated ≥2 fold in stumpy forms compared to slender forms. As shown for rodent-derived bloodstream forms, these reflect the fact that stumpy forms are cell-cycle arrested [24], with clear decreases in histone transcripts (Additional files 2 and 4). Tubulins and flagellar components are also down-regulated relative to slender forms. Once again this is consistent with the cells being quiescent, as the flagellum is only duplicated at the onset of mitosis [25]. Translation is also reduced in stumpy forms to about one-fifth of the rate in slender forms and there is a decrease in the number of polysomes [26]. Interestingly, transcripts for ribosomal proteins were not reduced significantly, but transcripts for the two versions of elongation factor 1A were down-regulated 2.5-fold and two Alba-domain proteins, Alba1 and Alba3 were reduced 3-fold. Alba proteins have previously been linked to initiation of translation in trypanosomes [27]. Transcripts encoding glycosomal proteins and glycolytic enzymes were also strongly down-regulated (Fig. 3b, Additional file 2), suggesting a reduced reliance on glucose as an energy source.

A large number of transcripts were more highly expressed in stumpy forms than in slender forms (Additional files 2, 4 and 5), probably reflecting pre-adaptation for transmission to the fly. This is reminiscent of a recent study of in vitro-derived metacyclic forms; these are cell-cycle arrested, and are poised to translate a bloodstream form proteome on transmission to the mammalian host [28]. GO term analysis [29] indicated a preponderance of transcripts in the signalling and cyclic nucleotide categories were significantly regulated (Additional file 2); all of these were adenylate cyclases. However, visual inspection revealed that 12 genes encoding components of inositol metabolism were also up-regulated (Table 1) [30–32]. These included all four target of rapamycin (TOR) orthologues [33, 34]. Depletion of TOR4 has previously shown to lead to the differentiation of monomorphic bloodstream forms to stumpy-like forms [34], so it is somewhat surprising that its expression is maximal in the latter.

**Table 1:** Genes implicated in inositol metabolism are up-regulated in stumpy forms

Gene ID	Description	St/Sl	St/Ea	Reference
Tb927.10.3890	Phosphatidylinositol-4-phosphate 5-kinase, putative (PIP5KII-alpha)	1.9	1.36	
Tb927.11.15330	Phosphatidylinositol 3-kinase catalytic subunit, putative	1.97	1.46	
Tb927.10.7110	Inositol-3-phosphate synthase	1.98	1.63	
Tb927.2.2260	Phosphatidylinositol kinase related protein, putative	2.53	0.78	
Tb927.10.8420	Target of rapamycin kinase 1 (TOR1)	1.83	1.04	
Tb927.4.420	Phosphatidylinositol 3-kinase (TOR2)	2.61	1.82	[33]
Tb927.4.800	Target of rapamycin kinase 3, putative (TOR3)	1.91	0.99	[33]
Tb927.1.1930	Phosphatidylinositol 3-kinase, putative (TOR4)	3.7	1.64	
Tb927.8.2770	Inositol 1,4,5-trisphosphate receptor (IP3R)	2.63	0.66	[30]
Tb927.7.4400	Inositol hexakisphosphate kinase	3.11	1.73	[31]
Tb927.8.3410	Inositol hexakisphosphate, putative	3.83	2.39	
Tb927.7.960	CMGC/SRPK protein kinase, putative (inositol pyrophosphate biosynthesis)	2.44	0.99	[32]

*St/Sl* fold change stumpy/slender forms, *St/Ea* fold change stumpy/early procyclic forms

In total, 267 genes were up-regulated ≥2 fold; these included PAD family members, ZC3H11 and squalene monooxygenase, all of which are up-regulated in animal-derived stumpy forms [35]. Kinetoplast-specific dual phosphatase (Tb927.7.7160), which was identified in an RNAi screen as driving stumpy formation, was also up-regulated 3.5-fold in stumpy forms. Other genes identified in the same RNAi screen, MEK kinase (Tb927.2.2720; 2.7-fold), Dyrk/YAK kinase (Tb927.10.15020; 1.9-fold) and two hypothetical proteins (Tb927.11.6600; 2-fold, and Tb927.9.4080; 3.5-fold), were found in biological replicate 1 only [36]. When analysed for peak expression in stumpy forms, 118 transcripts showed ≥2-fold more expression than in any other life-cycle stage (Additional file 5). These included zinc finger domain-containing proteins (ZC3H11, ZC3H 13, ZC3H20 and ZC3H32), several ubiquitin ligases, ubiquitin hydrolases, cyclin-like F-box protein 2 (CFB2) and a putative cyclin. Additionally, two kinases implicated as negative regulators of differentiation to procyclic forms, RDK1 and RDK2 [37], were most highly expressed in stumpy forms. Approximately one third of the transcripts encoded hypothetical proteins.

### Differential gene expression in early and late procyclic culture forms

As previously documented, the transition from the mammal to the tsetse fly is accompanied by major changes in morphology, metabolism and surface architecture [19, 38]. In total, 1245 genes were found to be differentially expressed ≥2-fold between stumpy and early procyclic forms. Many of these encode known surface proteins and components of the mitochondrion and glycosome. Apart from these, the transcripts most strongly up-regulated in procyclic forms are those for nitroreductase (Tb927.7.2980 and Tb927.7.3020; 50-fold) and two proteins with domains of unknown function (Tb927.11.7490 and Tb927.11.7500; 35- to 39-fold). These are also strongly up-regulated in the proteomes of differentiating and procyclic forms [39].

Previous work from our laboratory identified several proteins that were differentially expressed by early and late procyclic forms [6]. As shown in Additional file 4; DESeq analysis identified 73 transcripts that were differentially regulated between these life-cycle stages. Among them, 47 were up-regulated ≥2-fold in early procyclic forms, with GPEET being the most pronounced, and 26 were up-regulated in late procyclic forms. Several genes that were up-regulated in late procyclic forms appear to be specific for this particular stage, as they were also more highly expressed in tsetse-derived midgut forms 40 days post infection than in proventricular and salivary gland forms [8].

**Fig. 4:**
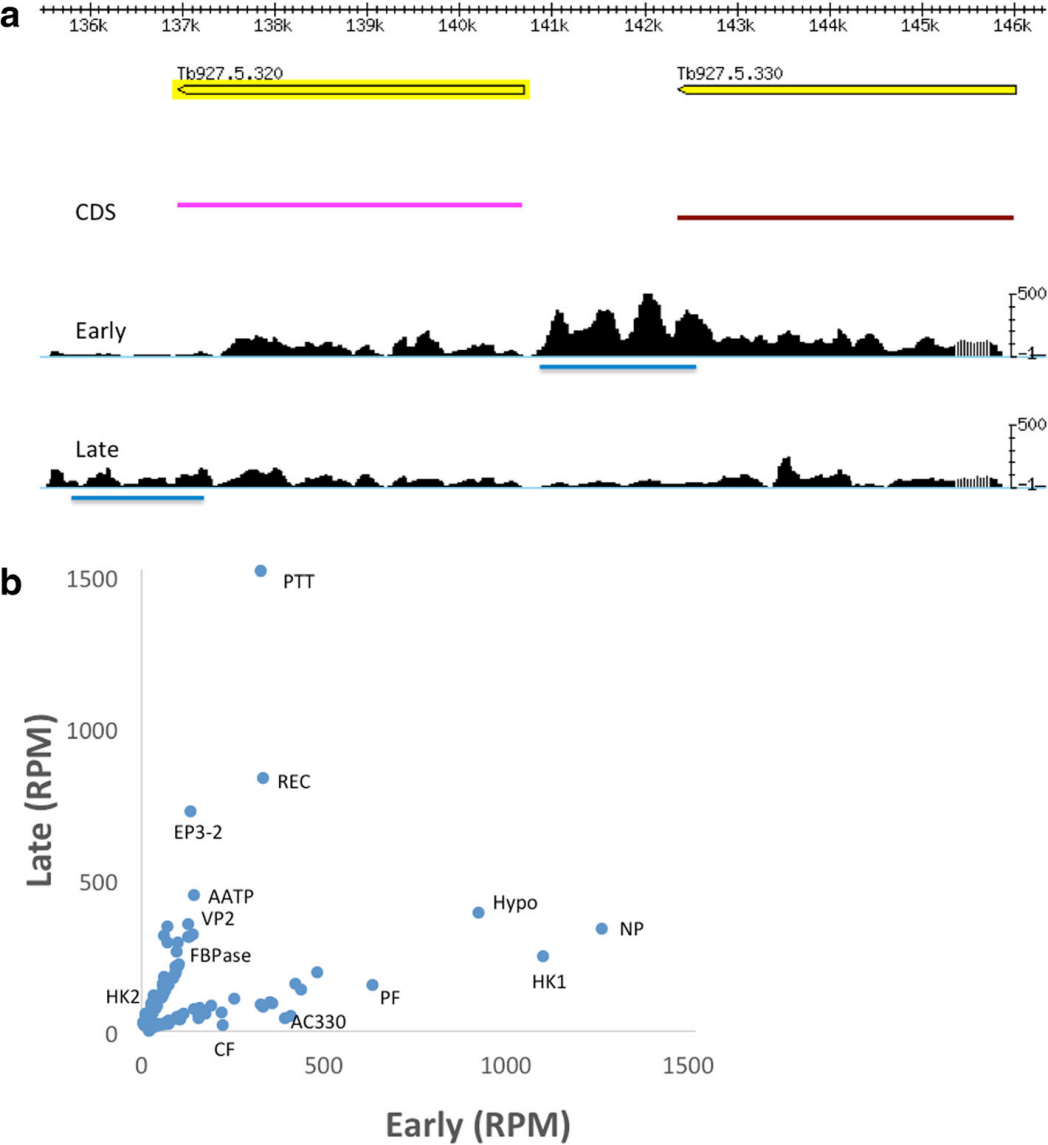
3′ untranslated regions allow discrimination between closely related coding sequences that are differentially expressed. **a** Gbrowse image showing mapping coverage of the locus encompassing the adenylate cyclases AC320 and AC330. 3’ UTRs are underlined in blue. Early: early procyclic forms; late: late procyclic forms. **b** Scatter plot of transcripts showing ≥2-fold differences between early and late procyclic forms, based on mapping to their 3’ UTRs. A comprehensive list is provided in Additional file 5. The scale is enlarged to show weakly expressed genes, resulting in the exclusion of GPEET. HK1: hexokinase 1; HK2: hexokinase 2; AC330, adenylate cyclase (Tb927.5.330); CF: calflagin (Tb927.8.5440); PTT, putative pteridine transporter; REC: RNA editing complex; Hypo: Hypothetical protein (Tb927.10.10000); EP3–2: EP procyclin; AATP: amino acid transporter (Tb927.11.6680); VP2: vacuolar proton pyrophosphatase 2 (Tb927.8.7980); FBPase: fructose-1,6-bisphosphatase (FBPase) (Tb927.9.8720); PF: prostaglandin f synthase (Tb927.11.4700); NP: nitrogen permease regulator 2 (Tb927.7.3010). RPM: reads per million

Many of the proteins that were shown to be differentially expressed by SILAC [6] reflected regulation at the level of mRNA (Table 2), including calflagins, prostaglandin F synthase, PTP1-interacting protein 39 (PIP39), pteridine reductase, adenylate cyclases and hexokinase 1 (HK1). HK1 and HK2 are virtually identical in their coding sequences, but differ in their 3’ UTR sequences. In addition, receptor-type adenylate cyclases Tb927.5.330 (AC330) and Tb927.5.320 (AC320) have extremely similar coding sequences but can be distinguished by their 3’ UTRs. Mapping coverage to 3’ UTRs was taken into account to identify the differential regulation of these genes between early and late procyclic forms (Fig. 4a). As shown in Additional file 6 and Fig. 4b, HK1 is up-regulated in early procyclic forms whereas HK2 is increased in late procyclic forms.

**Table 2:** Differential abundance of proteins (SILAC) and transcripts (RNA-Seq) in early and late procyclic forms

Up in early procyclic forms					
Gene ID	Description	SILAC rep1	SILAC rep2	RNA-seq Rep1	RNA-seq Rep2
Tb927.8.5460	Calflagin (flagellar calcium-binding protein 44)	8.4	3.59	5.75	5.01
Tb927.8.2210	Pteridine reductase (PTR1)	3.07	ND	1.96	2.12
Tb927.10.3660	Aspartate aminotransferase	3.26	3.18	2.35	2.95
Tb927.10.2010	Hexokinase (HK1)	3.4	2.93	4.48	3.74
Tb927.11.4700	Prostaglandin f synthase	2.37	2.4	4.94	3.81
Tb927.9.7000	Methyltransferase domain containing protein, putative	2.61	2.93	2.54	2.24
Tb927.9.6090	PTP1-interacting protein, 39 kDa	2.74	2.28	2.40	2.10
Tb927.1.2100	Calpain-like protein 1.1 (CALP1.1)	1.09	2.03	2.70	2.60
Tb927.7.7500	Thymine-7-hydroxylase, putative (TLP7)	4.08	1.97	3.52	2.44
Up in late procyclic forms					
Gene ID	Description	SILAC rep1	SILAC rep2	RNA-seq Rep1	RNA-seq Rep2
Tb927.1.3950	ALAT	2.44	1.68	3.45	0.85
Tb927.9.8720	FBPase	2.61	3.29	2.78	2.94
Tb927.7.190	Metallo-peptidase	2.77	2.58	1.40	1.67
Tb927.11.2500	Carboxypeptidase	2.46	2.72	2.13	1.79
Tb927.11.16550	Zinc finger protein, C3H1 type-like (ZC3H46)	8.78	33.24	1.0	3.03
Tb927.9.4200	Fatty acyl CoA synthetase 2 (ACS2)	2.19	2.07	1.43	1.70
Tb927.10.14140	Pyruvate kinase 1 (PYK1)	2.38	2.00	1.41	1.40

Numbers denote fold changes. Two biological replicates were performed in each case. The data for SILAC are derived from Imhof et al. [6]. *ND* not detected

Transcripts that are up-regulated ≥2 fold in late procyclic compared to early procyclic forms include fructose-1,6-bisphosphatase (FBPase; 2.6-fold), a cluster of putative pteridine transporters (4-fold), citrate synthase and aconitase [38]. FBPase and ACO proteins were previously shown to be up-regulated in late procyclic forms [6]. Receptor-type adenylate cyclases are another category showing differential regulation between early and late procyclic forms (Additional file 3). In addition to the reciprocally regulated neighbouring genes Tb927.5.320 (AC320) and Tb927.5.330 (AC330) [6], it is interesting to note that three other adenylate cyclases, ACP1, ACP4 and ACP6 are up-regulated in late procyclic forms. This was also reflected as the only significantly regulated group in the GO term analysis (Additional file 2). It was shown recently by Lopez and coworkers [40] that knock-down of ACP1 /ACP2 or ACP6 results in a hyper-SoMo phenotype. Since SoMo is a property of early procyclic forms [6], this suggests that depletion of these ACPs tilts the balance back towards this life-cycle stage.

### Individual members of multigene families show stage-specific expression

**Fig. 5:**
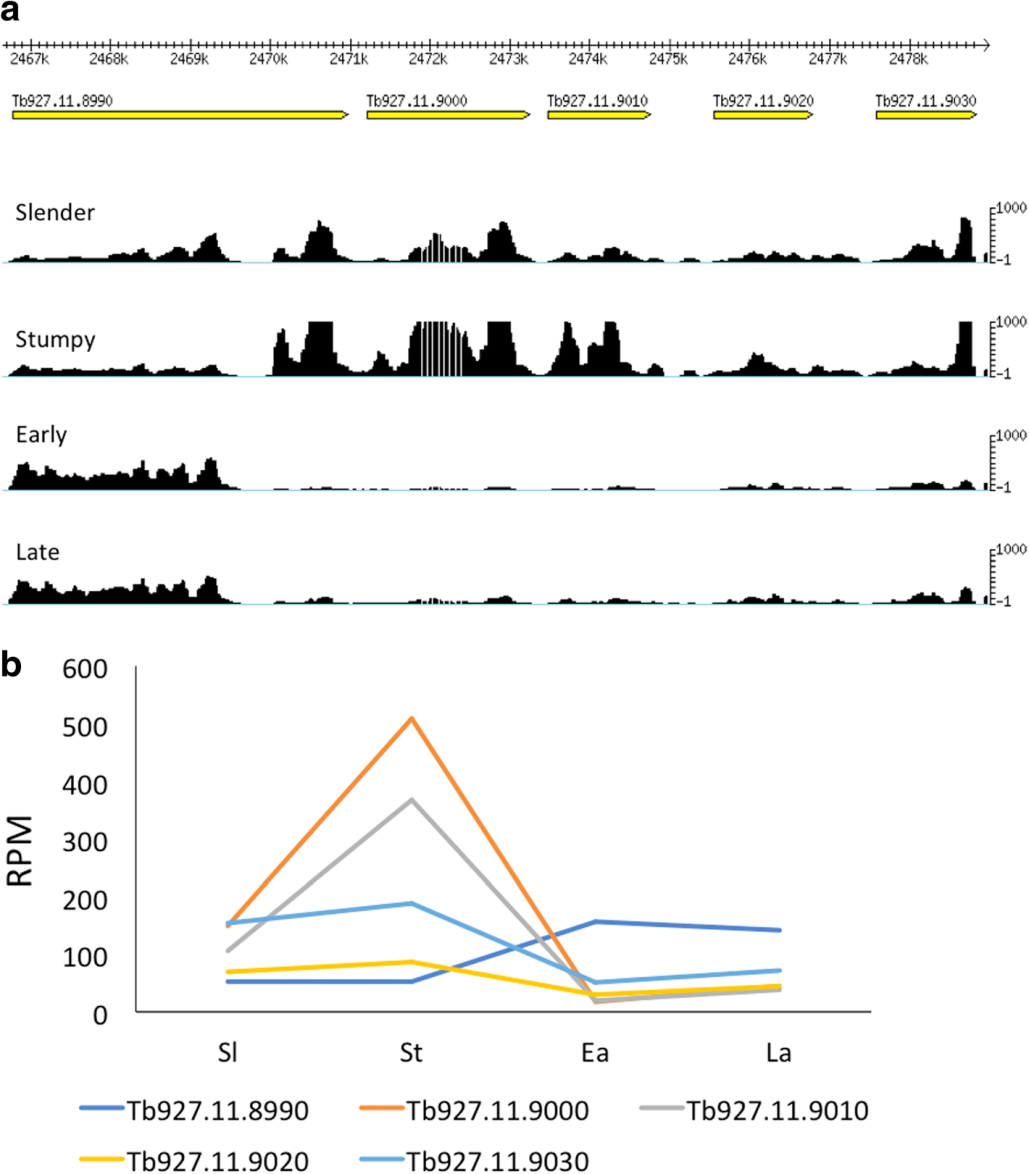
Cluster of cation transporters showing differential expression. **a** Gbrowse image showing mapping coverage of the locus. **b** Expression profiles of individual genes in successive life-cycle stages. Y-axis, reads per million (RPM). Sl: slender bloodstream forms; St: stumpy bloodstream forms; Ea: early procyclic forms; La: late procyclic forms

**Fig. 6:**
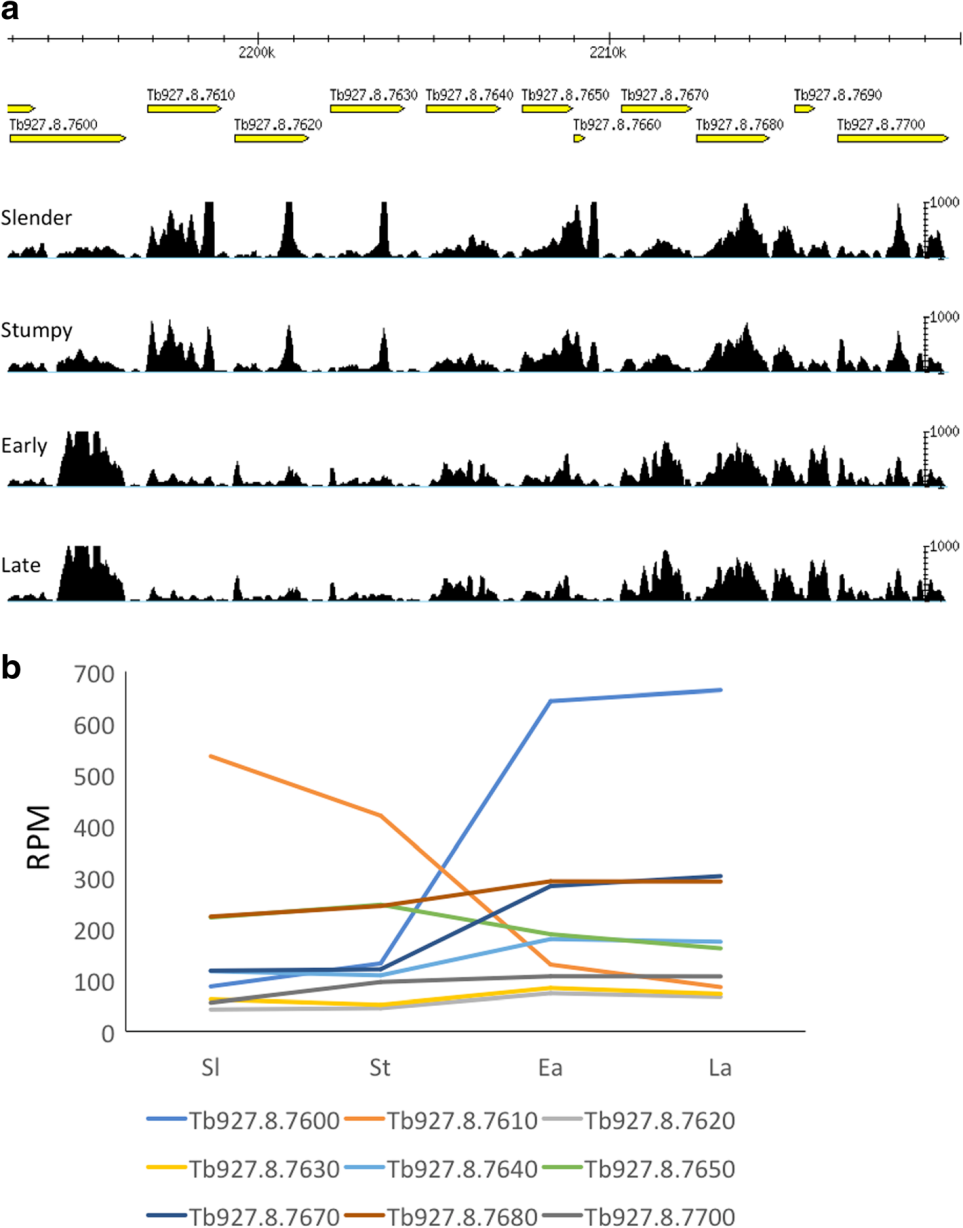
Cluster of amino acid transporters showing differential expression. **a** Gbrowse image showing mapping coverage of the locus. **b** Expression profiles of individual genes in successive life-cycle stages. Y-axis, reads per million (RPM). The coding region of Tb927.8.7610 is ≥96% identical to Tb927.8.7630/7640, but their expression profiles are distinct. Sl: slender bloodstream forms; St: stumpy bloodstream forms; Ea: early procyclic forms; La: late procyclic forms

With longer sequences, it is possible to assign reads to individual members of multigene families and demonstrate stage-specificity, even when coding regions share ≥96% identity. Of a cluster of 5 cation transporter genes on chromosome 11 (Tb927.11.8990–9030; Fig. 5), Tb927.11.8990 shows maximum expression in procyclic forms (both early and late), whereas Tb.927.11.9000 and 9010 show maximum expression in stumpy forms. The remaining two copies are expressed at low levels in bloodstream and procyclic forms, but are up-regulated in the salivary glands [8]. Likewise, a cluster of 9 amino acid transporter genes on chromosome 8 (Tb927.8.7600–7700) show differential expression. For example, Tb927.8.7610 and 7650 are most highly expressed in bloodstream forms and Tb927.8.7600 is most highly expressed in procyclic forms (Fig. 6). Tb927.8.7640, which is 96% identical to Tb.927.8.7610/7620/7630, is expressed at moderate levels in all 4 life-cycle stages that we analysed, but is up-regulated in the salivary glands, together with Tb927.8.7610 [8].

## Discussion

We have obtained comprehensive transcriptome data from cultures of four different life-cycle stages. It is highly encouraging that the expression profiles of all known stage-regulated genes identified in previous studies using different parasite strains, different sources and different methods are confirmed in our analysis. We conclude that slender and stumpy forms cultured in the presence of methylcellulose are excellent substitutes for parasites isolated from animals. Furthermore, this study, which provides the first RNA-Seq analysis of the transcriptome of stumpy forms, shows that many more genes are stage-regulated than was previously realised, with genes involved in inositol metabolism taking a prominent place. A number of genes that show peak expression in stumpy forms are expressed at similar levels in long slender and procyclic forms, and would therefore have been missed in earlier analyses. Our findings also underline that stumpy forms are not merely non-dividing bloodstream forms with some degree of pre-adaptation for transmission to tsetse, but are likely to have unique functions in the mammalian host. A first comparison of the transcriptomes of early and late procyclic forms shows that these are more closely related to each other than to other life-cycle stages, but they are clearly distinct. Most of the differential regulation of proteins described in a previous study of early and late procyclic forms [6] can be attributed to differences in steady stage mRNA, suggesting that translational control plays a relatively minor role at this point. When the analysis was extended to ~ 1200 proteins identified in the 2 SILAC datasets, the overall correlation coefficients (fold changes RNA:fold changes protein) were 0.46 and 0.59, respectively [6]. Comparing our RNA-seq data to the proteomics data sets from Dejung et al., [39] the correlation for RNA:protein in slender bloodstream forms was in the same range, at 0.48, while the correlation for stumpy forms was only 0.28. This is likely to reflect RNAs that are present, but not translated until the parasites begin to differentiate to procyclic forms. We could not perform a comparison between RNA and proteome for procyclic forms as the data from Dejung et al. [39] does not specify if their cultures are early or late procyclic forms (based on various markers we suspect that they are a mixture of the two). However, of 99 proteins down-regulated 24-48 h after triggering differentiation from stumpy to procyclic forms, 87 mRNAs were down-regulated in early procyclic forms.

In addition to providing new markers for all four life-cycle stages, these data also offer clues about metabolism. For example, genes encoding glycerol-uptake proteins are upregulated in stumpy forms, while glycerol kinases are upregulated in early procyclic forms. Unexpectedly, the THT2 hexose transporters are transiently upregulated in early procyclic forms. This may reflect a need for active acquisition of glucose in a sugar-poor environment, the insect midgut, and provide a window for maturation of the mitochondrion. Differentially regulated ion transporters and amino acid transporters presumably allow the parasites to sense and respond to their environment. Finally, the discovery of a relatively small number of differentially regulated genes between early and late procyclic forms may enable us to elucidate the signals and mechanisms involved in SoMo.

## Conclusions

This study provides the first transcriptomic data from cultures of four consecutive life-cycle stages of *Trypanosoma brucei*. As well as validating the use of cultured slender and stumpy bloodstream forms as alternatives to animal-derived parasites, in compliance with 3R principles, it provides the first comparison of the transcriptomes of early and procyclic forms and identifies new stage-regulated transcripts. Long reads enabled us to distinguish between closely related members of multigene families, and show that these are differentially expressed during the life cycle. Finally, this study delivers insights into the metabolic activities of the different life-cycle stages.

## Additional files

Additional file 1: Summary of mapping information. (DOCX 45 kb)

Additional file 2: Mean expression values (reads per million) for all transcripts. Sl: slender bloodstream forms; St: stumpy bloodstream forms; Ea: early procyclic forms; La: late procyclic forms. (XLSX 762 kb)

Additional file 3: GO term analysis of differentially expressed transcripts. (XLSX 83 kb)

Additional file 4: Differentially expressed genes (≥ 2-fold) in successive life-cycle stages. Sl: slender bloodstream forms; St: stumpy bloodstream forms; Ea: early procyclic forms; La: late procyclic forms. (XLSX 5584 kb)

Additional file 5: Stage-specific transcripts enriched ≥2-fold compared to all other stages. Sl: slender bloodstream forms; St: stumpy bloodstream forms; Ea: early procyclic forms; La: late procyclic forms. FC: fold change. (XLSX 59 kb)

Additional file 6: Differential expression ≥2-fold in early and late procyclic forms based on 3′ untranslated regions. Ea: early procyclic forms; La: late procyclic forms. (XLSX 44 kb)

## Abbreviations

Ea: Early procyclic forms
La: Late procyclic forms
RPM: Reads per million
Sl: Slender bloodstream forms
SoMo: Social motility
St: Stumpy bloodstream forms
UTR: Untranslated region
