# Supplementary material for: RNA-Seq analysis validates the use of culture-derived Trypanosoma brucei and provides new markers for mammalian and insect life-cycle stages
Source: BMC Genomics. 2018 Apr 2;19:227. doi: 10.1186/s12864-018-4600-6 (PMC5879877; doi:10.1186/s12864-018-4600-6)
Supplement: Supplementary file 1 — Summary of mapping information. (DOCX 45 kb) [file 12864_2018_4600_MOESM1_ESM.docx]

| **Sample** | **Total reads** | **Mapped reads** | **Mapping (%)** |
| --- | --- | --- | --- |
| Slender rep1 | 52,546,742 | 32,702,301 | 62.23 |
| Slender rep2 | 46,528,561 | 28,209,109 | 60.63 |
| Stumpy rep1 | 59,241,003 | 38,756,679 | 65.42 |
| Stumpy rep2 | 43,428,797 | 26,329,723 | 60.63 |
| Early rep1 | 40,123,774 | 32,774,250 | 81.68 |
| Early rep2 | 51,731,501 | 36,283,315 | 70.14 |
| Late rep1 | 41,119,450 | 33,687,334 | 81.93 |
| Late rep2 | 40,112,537 | 25,601,318 | 63.82 |

Mapping information
